# Interictal Fast Ripples Are Associated With the Seizure-Generating Lesion in Patients With Dual Pathology

**DOI:** 10.3389/fneur.2020.573975

**Published:** 2020-09-30

**Authors:** Jan Schönberger, Charlotte Huber, Daniel Lachner-Piza, Kerstin Alexandra Klotz, Matthias Dümpelmann, Andreas Schulze-Bonhage, Julia Jacobs

**Affiliations:** ^1^Epilepsy Center, Medical Center, University of Freiburg, Freiburg, Germany; ^2^Department of Neuropediatrics and Muscle Disorders, Medical Center, University of Freiburg, Freiburg, Germany; ^3^Faculty of Medicine, University of Freiburg, Freiburg, Germany; ^4^Berta-Ottenstein-Programme, Faculty of Medicine, University of Freiburg, Freiburg, Germany; ^5^Department of Paediatrics and Department of Neuroscience, Cumming School of Medicine, University of Calgary, Calgary, AB, Canada; ^6^Hotchkiss Brain Institute and Alberta Children's Hospital Research Institute, University of Calgary, Calgary, AB, Canada

**Keywords:** epilepsy, dual pathology, stereotactic electroencephalography, interictal, high-frequency oscillations, fast ripples

## Abstract

**Rationale:** Patients with dual pathology have two potentially epileptogenic lesions: One in the hippocampus and one in the neocortex. If epilepsy surgery is considered, stereotactic electroencephalography (SEEG) may reveal which of the lesions is seizure-generating, but frequently, some uncertainty remains. We aimed to investigate whether interictal high-frequency oscillations (HFOs), which are a promising biomarker of epileptogenicity, are associated with the primary focus.

**Methods:** We retrospectively analyzed 16 patients with dual pathology. They were grouped according to their seizure-generating lesion, as suggested by ictal SEEG. An automated detector was applied to identify interictal epileptic spikes, ripples (80–250 Hz), ripples co-occurring with spikes (IES-ripples) and fast ripples (250–500 Hz). We computed a ratio R to obtain an indicator of whether rates were higher in the hippocampal lesion (R close to 1), higher in the neocortical lesion (R close to −1), or more or less similar (R close to 0).

**Results:** Spike and HFO rates were higher in the hippocampal than in the neocortical lesion (*p* < 0.001), particularly in seizure onset zone channels. Seizures originated exclusively in the hippocampus in 5 patients (group 1), in both lesions in 7 patients (group 2), and exclusively in the neocortex in 4 patients (group 3). We found a significant correlation between the patients' primary focus and the ratio R_fast ripples_, i.e., the proportion of interictal fast ripples detected in this lesion (*p* < 0.05). No such correlation was observed for interictal epileptic spikes (*p* = 0.69), ripples (*p* = 0.60), and IES-ripples (*p* = 0.54). In retrospect, interictal fast ripples would have correctly “predicted” the primary focus in 69% of our patients (*p* < 0.01).

**Conclusions:** We report a correlation between interictal fast ripple rate and the primary focus, which was not found for epileptic spikes. Fast ripple analysis could provide helpful information for generating a hypothesis on seizure-generating networks, especially in cases with few or no recorded seizures.

## Introduction

Temporal lobe epilepsy is the most frequent cause for drug-resistant seizures (1). These patients have a higher chance of achieving seizure freedom if treated by epilepsy surgery rather than prolonged medical therapy (2, 3) and surgical outcomes are better if imaging revealed a potentially epileptogenic lesion (4, 5). Some individuals, however, have two lesions: One in the hippocampus and another one in the neocortex. In these “dual pathology” (6) patients, it is often unclear which lesion is seizure-generating, or if both lesions have such potential. Stereotactic electroencephalography (SEEG) may be helpful, but especially if only few seizures were captured, remaining uncertainty is considerable (7)—and patients rarely become seizure-free (1).

Even more in such scenarios, analysis of interictal activity may contribute substantially to presurgical evaluation. Most clinicians have focused on interictal epileptic spikes for decades and resection of spike-generating tissue correlates to some degree with post-surgical outcome in neocortical epilepsy (8). More recent studies suggest that high-frequency oscillations (HFOs), divided into ripples (80–250 Hz) and fast ripples (250–500 Hz), might have additional value when it comes to understanding epileptic networks and identifying epileptic foci. Resection of HFO-generating areas was associated with seizure-free outcome in several collectives (9–12), their rates increased after reduction of antiepileptic medication (13) and they may be involved in seizure generation (14–17). Many key studies on HFOs relied on visual identification, which is extremely time-consuming. During the past years, however, several automatic detectors have been developed (18–22). These tools now enable us to analyze HFOs in a clinical routine setting.

In this study, we hypothesized that interictal HFOs are associated with the seizure-generating lesion in patients with dual pathology. We applied an automated detector, compared spike, and HFO rates between the two lesions and examined whether this ratio correlates with the primary focus, as identified by ictal SEEG. Finally, we reviewed individual patients to estimate the value of our tool for clinical decision-making.

## Methods

### Patient Selection

We considered all patients with drug-resistant temporal lobe epilepsy who, as part of their evaluation for epilepsy surgery, had undergone stereotactic electroencephalography (SEEG) recordings at the Freiburg Epilepsy Center between 2012 and 2019. From these, subjects with two potentially epileptogenic lesions on neuroimaging were selected. All our patients had one lesion in the hippocampus and the other one in the temporal neocortex on the same side. In a few patients, radiologic findings were equivocal or only suggestive of a lesion. From these, we only included subjects with a lesion confirmed by histology. This study was approved by the Ethics Commission at the University Medical Center Freiburg and written informed consent was obtained from all patients.

### Grouping of Patients

**Figure 1:**
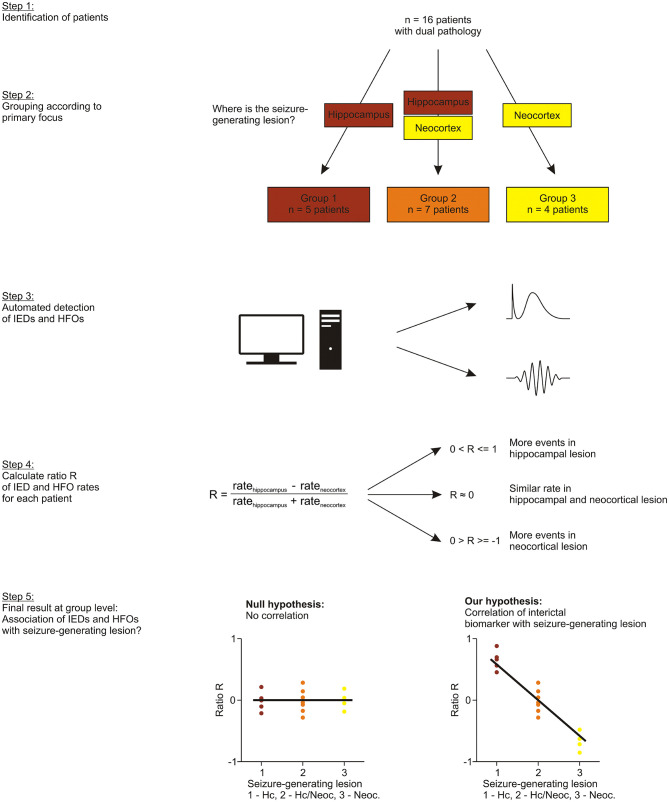
Study design. Patients with dual pathology were identified (step 1) and grouped according their seizure-generating lesion, as revealed by ictal SEEG (step 2). We then performed automated detection of interictal spikes and HFOs (step 3) and computed a ratio of rates R to obtain an indicator of whether events were more frequent in the hippocampal lesion (R close to 1), more or less similar (R close to 0) or more frequent in the neocortical lesion (R close to −1) (step 4). Finally, we examined if this ratio R, i.e., occurrence of our interictal biomarkers, was associated with the group that the patients had been assigned to, i.e., their seizure-generating lesion (step 5).

Depth electrodes (Ad-Tech Medical Instrument Corporation, Racine, WI) had been implanted based on their estimated value for clinical decision-making. Electrode contacts located inside the hippocampal or neocortical lesion were identified based on post-implantation MRI. We grouped our patients according to their seizure-generating lesion (Figure 1):

- Group 1: All recorded seizures generated in the hippocampal lesion
- Group 2: Some seizures generated in the hippocampal and some in the neocortical lesion, or onset more or less simultaneous in the two lesions
- Group 3: All recorded seizures generated in the neocortical lesion.

Grouping was performed based on our patients' medical reports only. Thus, regarding the decision of whether a seizure originated from the hippocampus or neocortex, we relied on the assessment of a board-certified neurologist who was blind to the purpose of this study.

### Interictal SEEG Data

SEEG was recorded with a Neuvo system (Compumedics, Abbotsford, Victoria, Australia). The sampling rate was 2 kHz and a low-pass filter with 800 Hz cut-off frequency was applied. For each patient, we selected a 1-h segment of slow-wave sleep, at least 2 h before and after a seizure. To determine if a contact was considered part of the seizure onset zone (SOZ), or not (non-SOZ), we used the judgement the independent clinical neurophysiologist made at the time of recording and clinical decision making.

### Detection of Interictal Epileptic Spikes and HFOs

We applied a recently developed automatic detector (23) to determine the rates of interictal epileptic spikes (IES), ripples (80–250 Hz), ripples co-occurring with spikes (IES-ripples), and fast ripples (250–500 Hz). This algorithm is based on a support vector machine, which is combined with a radial basis function kernel for non-linear classification. Simulated IES from a publicly available database (24) and visually identified HFOs were used for training. This detector has been tested against simulated and visually identified gold standards and, regarding HFOs, benchmarked against previously published algorithms. A detailed description of this method can be found in the original publication.

### Ratio R and R_fast ripples_ in Individual Patients

We computed a ratio R of mean rates (hippocampus—neocortex)/(hippocampus + neocortex) for each of these events. Thus, we obtained an indicator of whether

- events were more frequent in the hippocampal lesion (R close to 1)
- more or less similar in the two lesions (R close to 0) or
- more frequent in the neocortical lesion (R close to −1).

To explore the diagnostic value of fast ripple analysis in individual patients, those were finally ranked according to their R_fast ripples_. If R_fast ripples_ was an ideal biomarker, group 1 patients would have the top 5 values, group 3 patients the bottom 4 values, and group 2 patients would have values in between. For each subject, we thus determined retrospectively which primary focus might have been “predicted” as follows:

- R_fast ripples_ among top 5: Seizures generated exclusively in the hippocampal lesion (group 1)
- R_fast ripples_ among bottom 4: Seizures generated exclusively in the neocortical lesion (group 3)
- R_fast ripples_ in between (i.e., not among top 5 or bottom 4): Seizures generated in both lesions (group 2).

### Statistical Analysis

A significance level of 5% was chosen. The data was considered to be not normally distributed. We therefore specified the median as a measure of central tendency and the range as a measure of dispersion. The two-sided Mann-Whitney-*U*-test was applied to compare unpaired data. We performed Spearman's rank order correlation to examine the relationship between the group to which our patients had been assigned, i.e., their seizure-generating lesion, and the ratio R, i.e., the proportion of interictal epileptic spikes or HFOs detected in this lesion. These analyses were performed using SPSS (IBM, Armonk, NY).

A permutation test was conducted to examine whether R_fast ripples_ might have predicted the seizure-generating lesion in individual patients significantly better than chance [see e.g., (25, 26) for other examples of a permutation test]. To this end, we randomly shuffled the three group labels (5 × “1”, 7 × “2”, and 4 × “3”) between our 16 patients and then determined the number of correct “predictions,” which was between zero (no patient assigned correctly) and 16 (all patients assigned correctly). This procedure was repeated 100,000 times to compute a distribution of “surrogate” correct predictions. Finally, we compared our “empiric” number of correct predictions to this distribution to estimate the probability of obtaining such a result by chance. This part of our analysis was implemented in Matlab (Mathworks, Natick, MA).

## Results

### Patients and Their Seizure-Generating Lesions

We reviewed 115 patients with drug-resistant focal epilepsy who, as part of their evaluation for epilepsy surgery, had undergone SEEG recordings. Sixteen subjects (8 females, 8 males; age: median 39 years, range 12–53 years, see Table 1 for more clinical data) fulfilled inclusion criteria. The mesial temporal lesion was usually hippocampal sclerosis (*n* = 11), while the most frequent neocortical pathology was focal cortical dysplasia (*n* = 9) or a mild malformation of cortical development (*n* = 3). Most of our patients were treated by anterior temporal lobectomy, a minority received selective surgery of the hippocampal or neocortical lesion. We then grouped our patients according to their primarily seizure-generating lesion, as suggested by ictal SEEG: Seizures originated exclusively from the hippocampal lesion in five patients (group 1), from both hippocampus and neocortex in 7 patients (group 2) and exclusively from the neocortical lesion in four patients (group 3).

**Table 1:** Clinical data.

**ID**	**Hemisphere**	**Hippocampal lesion**	**Neocortical lesion**	**Surgery**	**12-month outcome**	**Seizure-generating**
					**(Engel class)**	**lesion (group)**
1	L	HS	FCD	/	/	1
2	R	HS	FCD	ATL	IIB	1
3	R	HS	Gliotic area/gray-white blurring	ATL	IA	2
4	R	HS	FCD	ATL	IA	1
5	L	Hc malformation	FCD	/	/	2
6	L	HS	FCD	EL + Hc resection	IB	2
7	R	HS	FCD	ATL	IIIA	2
8	R	HS	FCD	ATL	IIA^a^	1
9	R	HS	FCD	ATL	IA	3
10	R	HS	Gliotic area/gray-white blurring	ATL	IVB	2
11	L	Hc malformation	Meningoencephalocele	Temporal pole resection	IA	3
12	L	HS	Mild MCD	ATL	IA	1
13	R	Hc gliosis	FCD	ATL	IA	3
14	R	Hc gliosis	Meningoencephalocele	Temporal pole resection + AH	IA^a^	3
15	R	HS	Mild MCD	ATL	IA^b^	2
16	R	Hc gliosis	Mild MCD	ATL	IA^b^	2

*If 12-month outcome was not available, 3-month^a^ or 6-month^b^ outcome has been specified. AH, amygdalohippocampectomy; ATL, anterior temporal lobectomy; EL, extended lesionectomy; FCD, focal cortical dysplasia; Hc, hippocampus; HS, hippocampal sclerosis; L, left; R, right; y, years*.

### Spike and HFO Rates in Hippocampal vs. Neocortical Lesions

**Figure 2:**
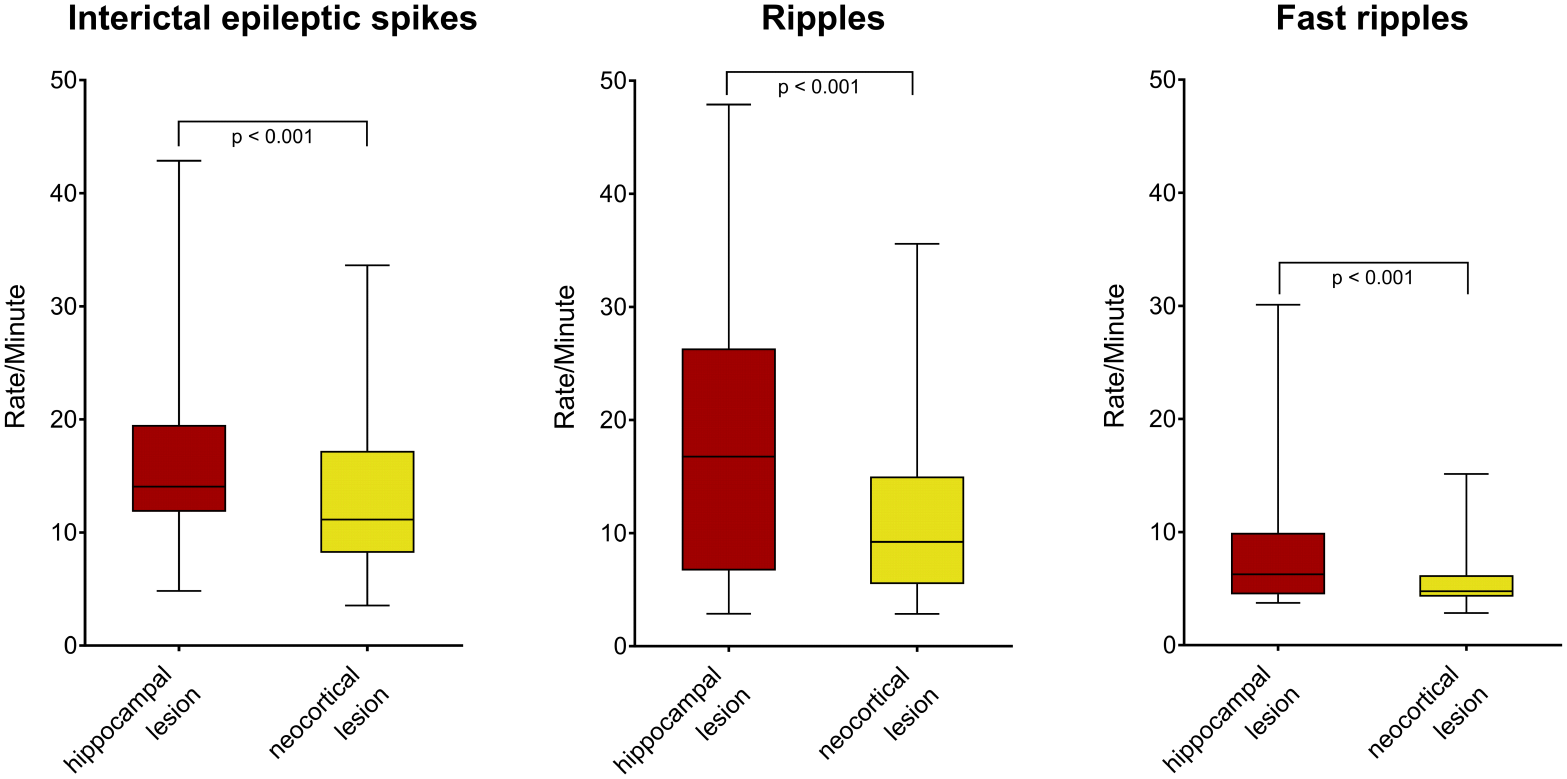
Rates of interictal spikes and HFOs in hippocampal vs. neocortical lesion. Note that spikes **(left)**, ripples **(middle)**, and fast ripples **(right)** occurred more often in channels located in the hippocampal lesion.

First, we compared the rates of interictal epileptic spikes and HFOs between the two lesions. Spikes, ripples, ripples co-occurring with spikes (IES-ripples) and fast ripples occurred significantly more often in electrode contacts located in the hippocampal lesion as compared to the neocortical lesion (Figure 2; *p* < 0.001; hippocampus: *n* = 60, neocortex: *n* = 124 channels; Mann-Whitney-*U*-test). When seizure onset zone (SOZ) and non-SOZ channels were analyzed separately, a significant difference was found inside the SOZ (Spikes: *p* < 0.05, ripples: *p* < 0.05, IES-ripples: *p* < 0.001, fast ripples: *p* < 0.01; hippocampus: *n* = 47, neocortex *n* = 46 channels; Mann-Whitney-*U*-test), but not for non-SOZ contacts (Spikes: *p* = 0.20, ripples: *p* = 0.93, IES-ripples: *p* = 0.61, fast ripples: *p* = 0.39; hippocampus: *n* = 13, neocortex *n* = 78 channels). Hippocampal lesions thus tend to generate more spikes and HFOs than neocortical lesions—and this difference seems to be specific to SOZ channels.

### Correlation of Spike and HFO Rates With Seizure-Generating Lesion

**Figure 3:**
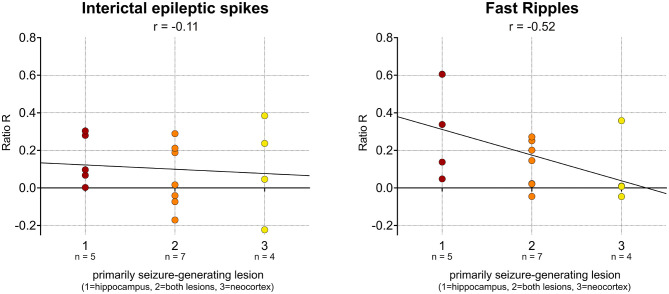
Association of interictal fast ripples with seizure-generating lesion. No significant correlation was observed for interictal spikes **(left)**. Note the significant correlation between ratio R, i.e., the proportion of fast ripples detected in a lesion, and the group that the patient had been assigned to, i.e., its seizure-generating potential **(right)**.

Keeping in mind this finding, it seemed rather unlikely that finding a higher spike or HFO rate in a patient's hippocampal lesion would indicate that this lesion also generates seizures. We therefore calculated the ratio R for each subject and examined if R, i.e., the proportion of spikes or HFOs detected in a lesion, correlates with the group to which the patient had been assigned, i.e., seizure genesis in this lesion. Such a correlation was found for interictal fast ripples (Figure 3; *r* = −0.52; *p* < 0.05; Spearman's rank order correlation), but not for spikes (*r* = −0.11; *p* = 0.69), ripples (*r* = −0.14; *p* = 0.60), or IES-ripples (*r* = −0.17; *p* = 0.54). Of note, these analyses were performed on interictal data from all electrode contacts located in either of the two lesions—thus, R was calculated independent from any information on the patient's seizures. In summary, our findings suggest that R_fast ripples_ is a biomarker which is specifically associated with the seizure-generating lesion.

### Diagnostic Value for Individual Patients

**Figure 4:**
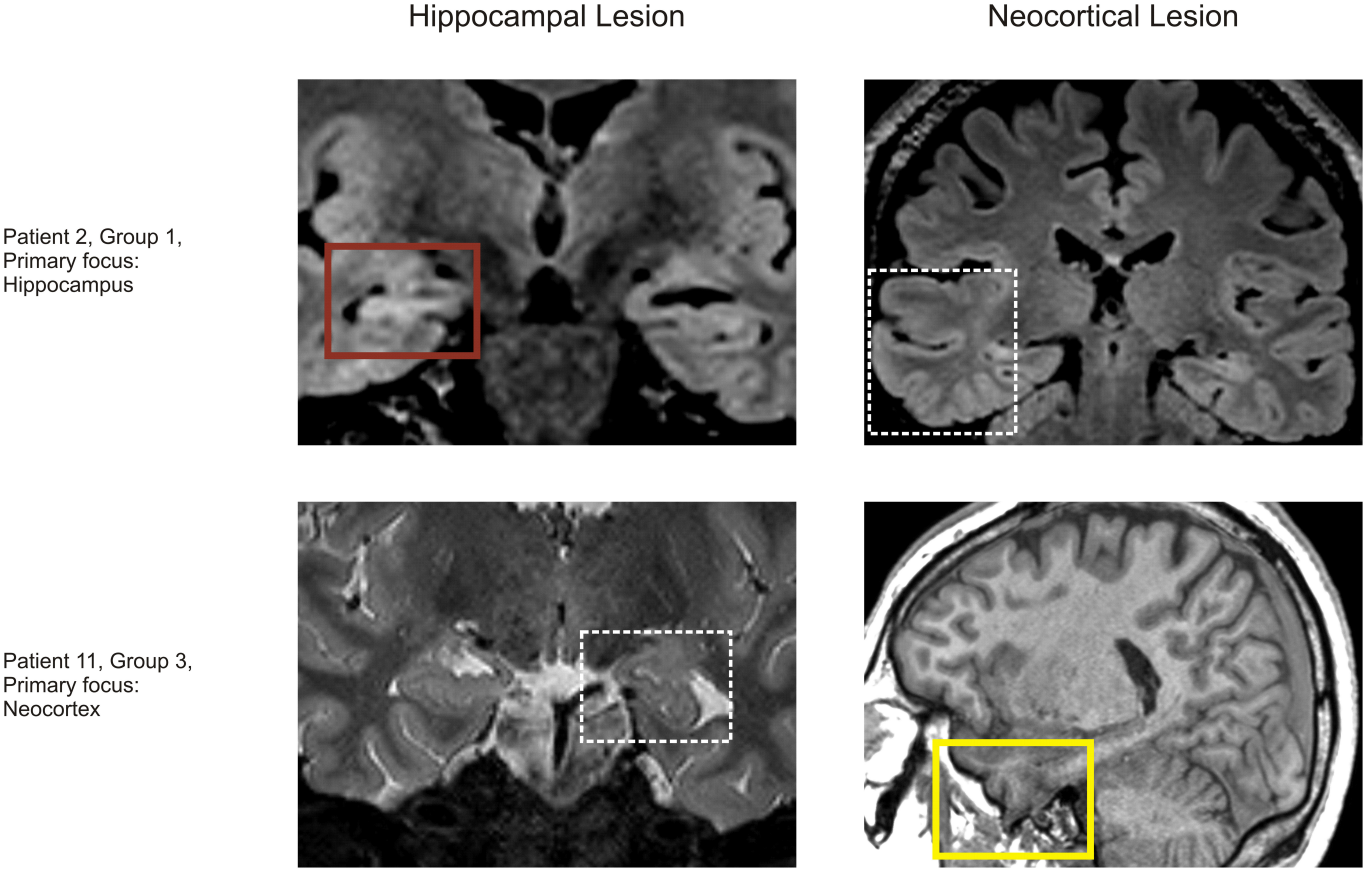
Two exemplary patients. (Upper row) Patient 2 had her primary focus in the hippocampal lesion. MRI showed hippocampal sclerosis (upper left) and a temporal lobe FCD (upper right, white box). Ictal SEEG suggested that seizures were only generated in the hippocampal lesion and interictal fast ripples were more frequent in the hippocampus (R = 0.61; red box). (Lower row) Patient 11 had his primary focus in the neocortical lesion. MRI showed a hippocampal malrotation (lower left, white box) and a temporal lobe meningoencephalocele (lower right). Ictal SEEG suggested that seizures were only generated in the neocortical lesion and interictal fast ripple rate was more or less similar (in this case slightly higher in the neocortical lesion; R = −0.05; yellow box).

Finally, we aimed to explore whether an analysis of interictal fast ripples could be of diagnostic value for individual patients. If fast ripples were a good biomarker, R_fast ripples_ would be high in most subjects with seizures originating from the hippocampal lesion and low in those with neocortical onset (Figure 4). As we retrospectively estimated performance by a data-based approach, we obtained correct “predictions” in 11 of our patients (69%; *p* < 0.01, permutation test; Table 2). Correct or incorrect predictions were not obviously linked to a distinct pathology. Thus, fast ripple analysis might classify above chance, but performance would be impaired due to the overlap between different groups.

**Table 2:** Interictal fast ripple analysis and seizure-generating lesion in individual patients.

**Ratio R_**fast ripples**_**	**Patient ID**	**Seizure-generating lesion (group), as revealed by ictal SEEG**	**Seizure-generating lesion (group), as “predicted” by ratio R_**fast ripples**_**	**Prediction correct?**
0.61	2	1	1	Yes
0.60	12	1	1	Yes
0.36	13	3	1	No
0.34	1	1	1	Yes
0.27	10	2	1	No
0.25	3	2	2	Yes
0.20	5	2	2	Yes
0.15	15	2	2	Yes
0.14	4	1	2	No
0.05	8	1	2	No
0.02	7	2	2	Yes
0.02	16	2	2	Yes
0.01	9	3	3	Yes
0.01	14	3	3	Yes
−0.05	6	2	3	No
−0.05	11	3	3	Yes

*To estimate the performance value of our tool, we ranked our subjects according to their ratio of rates R. For each individual, we then retrospectively determined if R_fast ripples_ would have correctly “predicted” the seizure-generating lesion. An ideal biomarker would sort group 1 patients to the top of this table, group 3 patients to its bottom and group 2 patients in between. 11 patients (69%) were assigned correctly (p < 0.01). See Methods section for a detailed description of our approach*.

## Discussion

The main novel findings of this study are that in patients with dual pathology (1) interictal spikes and HFOs are more frequent in the hippocampal pathology, particularly in seizure onset zone channels, (2) fast ripples are associated with the seizure-generating lesion, and (3) might have some diagnostic value for individual patients.

### Hippocampal Lesions Generate More Spikes and HFOs

We report that spike and HFO rates were higher in hippocampal than in neocortical lesions—and that this difference is specific to the seizure-onset zone (SOZ). This result is consistent with a previous study suggesting that HFOs are primarily an indicator of epileptogenicity (27, 28). Analyzing subjects with dual pathology, we could now directly compare biomarker occurrence between the two lesions. Most of our patients had hippocampal sclerosis and focal cortical dysplasia. Therefore, our results may not be representative of other pathologic entities, such as e.g., post-ischemic alterations or tumors. At the end, we can only speculate on the main reasons for which the seizure-generating portion of lesions in the hippocampus might generate more HFOs: Its complex architecture, with several distinct three-layered sub-regions, contrasts with six-layered neocortex in healthy individuals. Hippocampal sclerosis and focal cortical dysplasia are furthermore due to a fundamentally different pathogenesis. In some patients, it was hard to clearly delineate the neocortical lesion; it could thus be hypothesized that sometimes our electrodes did not record from tissue with maximum pathogenicity. Finally, the hippocampus is suited best for generation of physiological HFOs (29–31), and network alterations associated with epilepsy might exploit this machinery—a concept that has also been proposed e.g., for spike-wave seizures (32).

### Association Between Interictal Fast Ripples and Seizure-Generating Lesion

Since the hippocampus in general (31, 33), and hippocampal lesions in particular, tend to generate more HFOs than the neocortex, it is not trivial to compare the epileptogenicity of these two regions—observing slightly higher rates in the hippocampus e.g., does not indicate that this is the primary focus. Nevertheless, we report that in patients with dual pathology, the potential of a lesion to be seizure-generating correlates with its potential to generate fast ripples. This conclusion was based on calculations of the ratio R, which adjusts for the fact that hippocampal lesions have in general higher fast ripple rates. Such a correlation was not found for interictal spikes, ripples, or ripples co-occurring with spikes (IES-ripples). These findings are in line with previous work suggesting that HFOs might identify epileptogenic tissue better than spikes (9, 34). It is still subject to debate which of the HFO subgroups is suited best as a biomarker, but a popular notion is that ripples lack specificity, possibly because some of the events are physiological. One strategy to overcome this problem could be to analyze ripples associated with spikes, which may perform better in distinct clinical scenarios (23, 34). The other main approach has been to focus on fast (10) and very fast ripples (35, 36): Those might only rarely be physiological (31), thus be more specific, and also be involved in seizure generation (15, 16, 37). Our present study clearly supports this view of fast ripples as a biomarker with unique properties—at least in distinct clinical scenarios.

### Value for Clinical Decision-Making in Individual Patients

We report that two variables correlate at the group level. But from a clinician's point of view, the question is: Could this biomarker be useful for decision-making in individual patients? Presurgical workup in dual pathology aims to evaluate whether both lesions can generate seizures—if so, anterior temporal lobectomy is often recommended, whereas more restrictive surgery might be considered if concordant findings suggest that only one lesions has seizure-generating potential and even more if the second lesion is not clearly visible on MRI. Based on data obtained in this study, we estimated that interictal fast ripples might have correctly predicted the seizure-generating lesion in 69% of the patients. This approach permits only to crudely estimate the value of our tool, which seems to perform better than chance, but no better than traditional elements of presurgical evaluation. At present, HFOs are rarely studied in a clinical routine setting, but we hope that application of a publicly available detector will promote such analyses. In summary, interictal fast ripples could be considered to obtain complementary information on seizure-generating networks—especially in cases with few or no recorded seizures.

### Limitations and Outlook

The current study has some limitations and additional work is needed to fully investigate the role of HFOs in patients with dual pathology. A sample size of 16 subjects only permits to detect pronounced differences. Besides, our study is purely retrospective. Especially when it comes to estimating the value of fast ripples in individual patients, we would have needed more subjects for a thorough analysis and our tool might have performed worse if tested in another sample of patients. Finally, it should be considered that the reference to which we compared our HFO data was the seizure-generating lesion, as determined by SEEG, and not post-surgical outcome because most of our patients were treated by anterior temporal lobectomy. This implies that patients grouped as “hippocampal” or “neocortical” could have seizures originating from the other lesion that had just not been captured—or that, after resection of the primary focus, the “secondary” lesion might start to generate seizures. These limitations can only be overcome by a larger, if possible prospective, study that relates HFO data to post-surgical seizure outcome. Before we move on to this step, it may be interesting to analyze additional aspects of HFOs, e.g., the temporal relationship between events from the two lesions. Such an approach could not only yield a diagnostic tool for dual pathology—it might in general delineate the role of HFOs in epileptogenic networks further.

## Data Availability Statement

The data analyzed in this study is available on reasonable request. Requests to access these datasets should be directed to jan.schoenberger@yahoo.de.

## Ethics Statement

The studies involving human participants were reviewed and approved by Ethics Commission, University Medical Center Freiburg. Written informed consent to participate in this study was provided by the participants' legal guardian/next of kin.

## Author Contributions

JS and JJ designed the study and wrote the manuscript. JS, CH, DL-P, KK, MD, AS-B, and JJ acquired and analyzed data and edited and approved the final version of the manuscript. JS, CH, AS-B, and JJ drafted the figures and tables. All authors contributed to the article and approved the submitted version.

## Conflict of Interest

The authors declare that the research was conducted in the absence of any commercial or financial relationships that could be construed as a potential conflict of interest. The handling editor is currently organizing a Research Topic with one of the authors JJ.
